# Heterologous Expression of Extracellular Proteinase pAsPs of *Aspergillus pseudotamarii* in *Komagataella phaffii*

**DOI:** 10.3390/ijms232315035

**Published:** 2022-11-30

**Authors:** Andrey Valentinovich Zadorozhny, Mikhail Evgenyevich Voskoboev, Denis Vladimirovich Bochkov, Alexei Sergeyevich Rozanov, Elizaveta Dmitrievna Shedko, Irina Anatolyevna Mescheryakova, Alexander Gennadyevich Blinov, Anton Vladimirovich Korzhuk, Valeria Nikolayevna Shlyakhtun, Natalia Vladimirovna Bogacheva, Egor Vladimirovich Antonov, Svetlana Valerevna Bannikova, Tatiana Nikolayevna Goryachkovskaya, Sergey Evgenyevich Peltek

**Affiliations:** 1 Laboratory of Molecular Biotechnology, The Institute of Cytology and Genetics, SB RAS, 630090 Novosibirsk, Russia; 2 Kurchatov Genomic Center of the Institute of Cytology and Genetics, SB RAS, 630090 Novosibirsk, Russia

**Keywords:** proteinase, *Komagataella phaffii*, multicopy, heterologous expression, *Aspergillus pseudotamarii*, industrial application

## Abstract

Neutral protease pAsPs gene was obtained by sequence optimization of NpI protease from *Aspergillus pseudotamarii.* pAsPs was for the first time integrated in the genome of yeast strain *Komagataella phaffii* T07, and then produced in a 5 L bioreactor with an enzyme yield of 150,800 U/mL of culture liquid towards casein. The specific activity of the pAsPs was 7,657,000 U/mg toward casein, 2320 U/mg toward hemoglobin, and 25,344 U/mg toward azocasein per 1 mg of the protein. The enzyme was found to be inhibited by Cu^2+^. Optimal activity pH was shown in the range of pH 6.5–8.0, and optimal temperature—50–60 °C. The molecular mass of the recombinant protease pAsPs was shown to be 67.5 kDa. Mass-spectrometric analysis confirmed the identity of the amino acid sequence of the obtained pAsPs preparation with the predicted sequence, with 17% coverage and protein score 288. Thus, the novel neutral protease pAsPs is a promising candidate for large-scale use in manufacturing, including the food industry.

## 1. Introduction

Secreted fungal proteases are among the major enzymes in industry, including the pharmaceutical, detergent, and leather industries [1], as well as bioactive peptides and waste-processing–related manufacturing [2]. The global market for manufacturing associated enzymes in 2011 was estimated to be approximately $3.3 billion, but in 2020 in the US alone, it was shown to be $1.5 billion and predicted to continue growing at 7.1% per year until 2027 [3]. Meanwhile, fungal proteases account for 20% to 60% of the proportion of the total enzyme market [2]. Currently, such enzymes can be obtained either by their production directly in host organisms [4] or via heterologous expression in other organisms [5].

Proteinases (EC 3.4.23.X) are the enzymes hydrolyzing peptide chains with wide substrate specificity (Kamal). Proteinases belong to six different classes based on the mechanism it catalyzes: cysteine proteinases, serine proteases, asparagine proteases, metalloproteases, threonine proteases, and unknown types of proteases. Every class is defined by the specific number and configurations of amino acid residues in the active site [6]. Neutral proteases have high specificity for hydrophobic amino acid residues [7]. Enzymatic activity of fungal proteases is mediated by the formation of an intermediate acyl enzyme, which covalently attaches to the N terminus of a substrate, which is then hydrolyzed by a water molecule [2].

*Komagataella phaffii* (formerly known as *Pichia pastoris*) is widely used in fundamental research and for industrial recombinant proteins synthesis. *K. phaffii* has many advantages, such as a simple and inexpensive medium, high productivity, low native protein levels, and simplified protein purification [8,9]. Various proteases have been successfully expressed in *K. phaffii*, including a serine protease from *Trichoderma koningii* [8], an alkaline protease from *Aspergillus oryzae* [10], and a neutral protease from *Aspergillus oryze* [9].

Currently, the most common commercially available extracellular NpI proteinase is *Aspergillus oryzae* protease NpI; NpI can be produced in baculoviruses, mammalian cell lines, yeast, or *Escherichia coli*, whereas a recombinant NpI metalloproteinase has high levels of expression in *Komagataella phaffii* [9]. The NpI proteinase was shown to be the most prevalent proteolytic enzyme during soybean fermentation at late stages of soy sauce production [11]. Furthermore, neutral proteases are widely employed in the food industry to reduce the bitterness of foods and to control nitrogen levels [12].

*Aspergillus pseudotamarii* was discovered in 2001 as a new aflatoxin-producing isolate of *Aspergillus tamarii*, a member of the Flavi section in the Aspergillus genus. In addition to the synthesis of aflatoxin B, it has been reported that *A. pseudotamarii* fails to grow conidia at 42 °C, unlike *A. tamarii* [13]. The use of such microorganisms in industry is difficult due to toxicity; however, practical application of heterologous producer strains carrying genes from the fungal strains that are not GRAS (generally recognized as safe) is quite promising.

In this study we constructed a unique strain expressing the pAsPs protease gene from the fungus *A. pseudotamarii*, as well as optimized the conditions for lab-scale production (in a bioreactor) of the previously designed enzyme.

## 2. Results

### 2.1. Preparation of the Genetic Construct for Expression in K. phaffii T07

The K. phaffii T07 strain is the wild type of the *K. phaffii* species found in the territory of Sevastopol (Russia). It was discovered that the genotype of strain T07 is 99.96% similar to the strains *K. phaffii* CBS7435 (GCA_000223565.1) and *K. phaffii* GS115 (GCA_000027005.1). This analysis was performed in the CompareM software with the aai_wf option (https://github.com/dparks1134/CompareM, accessed on 23 March 2022).

Plasmid pPZL, previously obtained in our laboratory [14], was applied to create a plasmid with tandem copies—pPZL-2xProt_AsPs. This plasmid carries a tandem construct containing the optimized *A. pseudotamarii* protease gene under the control of the AOX1 gene promoter and the terminator for inducible expression in the yeast *K. phaffii*, as well as an origin of plasmid replication, a zeocin resistance gene under the control of the EM7 gene promoter for expression in E. coli, and the TEF1 gene promoter for expression in *K. phaffii*.

**Figure 1:**
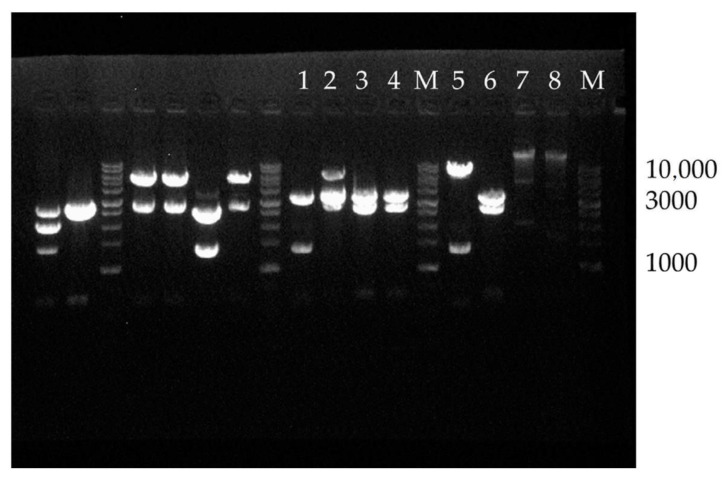
Electrophoresis of the plasmid carrying tandem copies of the target gene. Lanes 1,3,4,6—plasmids digested with *Sma*I, *Bgl*II, and *Bam*HI carrying one gene copy; 2—plasmid digested with *Sma*I, *Bgl*II, and *Bam*HI from the colonies carrying both plasmid variants; 5—plasmids digested with *Sma*I, *Bgl*II, and *Bam*HI carrying two gene copy; 7,8—colonies with incorrect plasmids; M: molecular weight markers with a maximum size of 10,000 bp.

After electroporation of the pPZL-2xProt_AsPs plasmid into *E. coli* cells, the colonies appeared on the plates were incubated in 4 mL of the LB medium with zeocin (20 μg/mL) at 37 °C and 250 rpm for 12 h. Then, according to the manufacturer’s instructions, plasmid DNA was isolated with the QIAprep Spin Miniprep Kit (Qiagen, Germany). The isolated DNA was digested with restriction enzymes SmaI, BglII, and BamHI. The presence of tandem copies of the pAsPs protease gene from *A. pseudotamarii* and the correct orientation of the components were verified by electrophoresis in a 1% agarose gel (Figure 1).

According to the results of the electrophoretic analysis, a clone (Figure 1, lane 5) carrying a plasmid of the correct composition was chosen, for the fragment after restriction was shown to be the correct length. The respective plasmid was subsequently subjected to electroporation into yeast strain *K. phaffii* T07.

The resulting *K. phaffii* transformants were used to produce the target protein in bioreactors.

### 2.2. Optimization of Production Parameters in the Bioreactor

**Figure 2:**
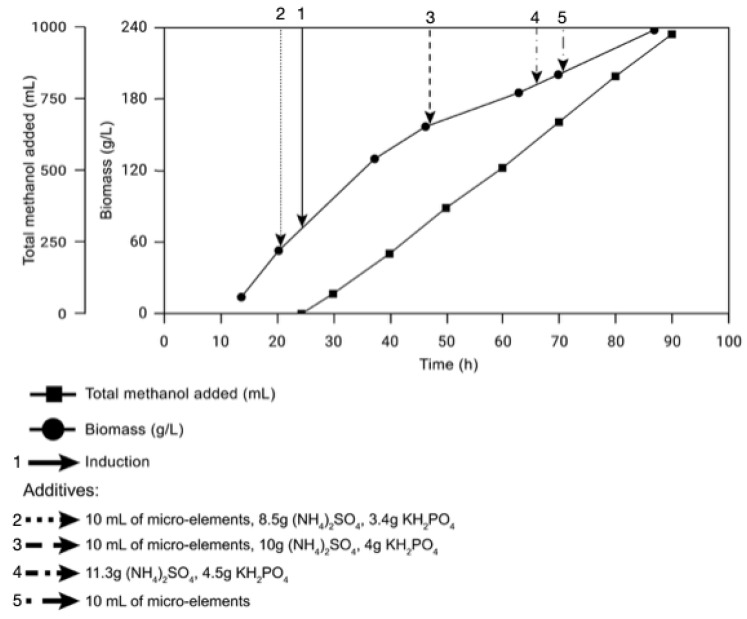
The graph of biomass growth of *K. phaffii* strain AsPs and the supply of methanol during the cultivation in the bioreactor.

It is known that *K. phaffii* biomass may amount to >100 g/L if methanol or another simple compound, such as glucose or glycerol, serves as a substrate [14]. After transformants were placed in the bioreactor and reached the biomass of 90 g of cell mass per liter of wet mass—along with a significant increase in the level of dissolved oxygen, indicating a decrease in the concentration of glycerol in the medium [11]—we proceeded to the induction stage. Next, 10 mL of trace elements and 10 g of (NH_4_)_2_SO_4_ were added into the bioreactor. A 60% methanol solution was used as an inducer of the AOX1 promoter. The induction step was started by lowering the temperature to 27 °C and addition of 40 mL inducer. After the oxygen level was reduced to 20% and methanol adaptation, 4 mL of methanol was added every 20 min in the first 3 h after the induction, and then 6.7 mL until the end of cultivation. If a sharp increase in dissolved oxygen above 25% was detected, then 40 mL of the inducer was added. The cultivation lasted for 3 days after the induction initiation. When a biomass content of 150 g/L was reached, the temperature was lowered to 23 °C. Microbial growth was seen throughout the fermentation process, with a maximum biomass content of 240 g/L (Figure 2).

### 2.3. Mass-Spectrometric Analysis

The activity of the culture liquid toward casein was found to be 150,800 U/mL, and the activity of the protein from the culture liquid toward casein proved to be 309,650 U/mg. Afterward, purification of the pAsPs protease on the DEAE-Sepharose 6HF anion exchange resin was performed. The resulting protein was 24.7-fold relative to the initial activity of the pAsPs protease in the culture liquid. The degree of purification in DEAE-Sepharose was 1.9-fold with respect to the purification in Sephadex G-25.

After chromatographic purification of pAsPs from the lyophilized culture liquid, a pAsPs sample was obtained with an activity of 7,657,000 U/(mg of protein) toward casein, 2320 U/(mg of protein) toward hemoglobin, and 25,344 U/(mg of protein) toward azocasein.

**Figure 3:**
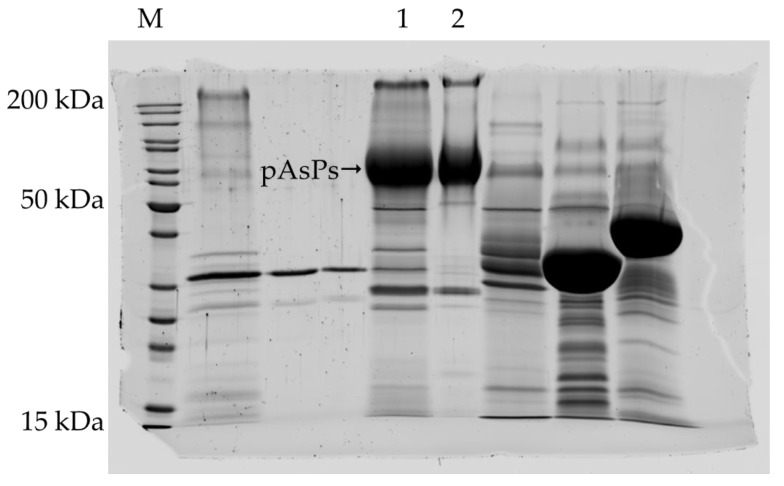
Electrophoretic protein analysis. 1:SDS-PAGE separation of proteins in the sample of protease pAsPs purified on Sephadex G-25; 2: SDS-PAGE separation of proteins in the sample of protease pAsPs purified by ion exchange chromatography. Molecular weight markers: PageRuler™ Unstained Protein Ladder (ThermoFisher).

The nominal molecular mass of the recombinant protease pAsPs was found to be 67,575 Da, which matches the calculated molecular mass: 67.6 kDa (Figure 3).

**Figure 4:**
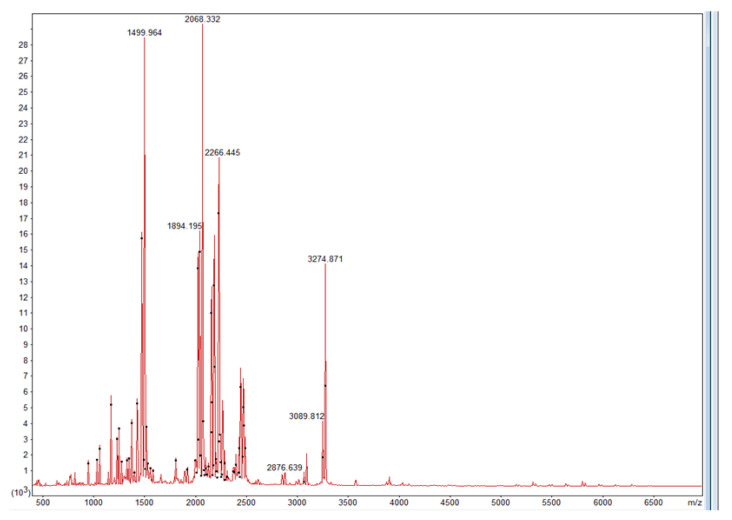
The mass spectrum of protease pAsPs.

The MS1 spectrum of protease pAsPs is shown in Figure 4.

The protein score (PS) is the sum of the ions score for each distinct sequence with a small correction that was shown to be 288, which is a statistically significant result (http://www.ohri.ca/proteomics/docs/understandingmascotreports.pdf, accessed on 5 April 2022), and the calculated isoelectric point is 4.86 [15]. The sequence coverage was 17%. Individual ions scores >52 indicate identity or extensive homology (*p* < 0.05) (Table 1). The analysis was performed using lyophilized culture liquid.

Therefore, authors imply, it be concluded that the neutral pAsPs protease was reliably identified.

### 2.4. Activity and Related Parameters of the Enzyme

**Figure 5:**
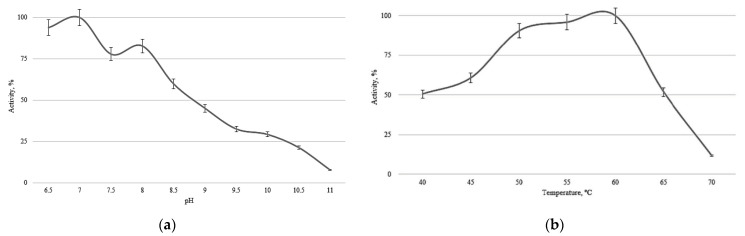
Protease pAsPs activity: pH levels (**a**) and temperatures (**b**).

The pH optimum of the pAsPs protease activity is 6.5–8.0, with the highest activity observed at pH 7 (Figure 5a). The temperature of activity optimum was 50–60 °C, with the highest activity at 60 °C (Figure 5b). Activity toward casein after chromatographic purification was taken as 100% during the experiments.

It was shown here that high concentrations of metal ions reduce the activity of the recombinant pAsPs protease to various degrees (Table 2), but only Cu^2+^ completely inhibits the enzyme.

### 2.5. Enzyme Stability under Optimal Conditions

The initial specific activity of the enzyme was found to be 8,085,714 U/mg. After 10 min of incubation, the enzymatic activity decreased to 78.8% of the initial value and amounted to 6,371,420 U/mg. After 20 min of incubation, the enzymatic activity was 2,285,715 U/mg, and after 30 min 742,850 U/mg. Testing of a sample taken 40 min after the start of incubation revealed complete inactivation of the enzyme.

## 3. Discussion

Strain *K. phaffii* T07, previously obtained in our laboratory [16], was shown to be highly similar to widely used strains, such as *K. phaffii* GS115 (having his4 mutations) and X-33 (which is a strain reverted to the wild type by site-directed mutagenesis) [17]. Since protease pAsPs gene was derived from the fungi, expression in *K. phaffii* provides an opportunity to minimise the effects of heterologus expression, such as posttranslation modification or inclusion bodies formation. In addition, *K. phaffii* is currently a widely used biological object, both as a model organism [18] and as a producer of biopolymers [19]; thus, the expression system of AsPs protease is suitable for further large-scale production. Hu et al. generated recombinant extracellular neutral protease of wild-type *A. oryzae* in *E. coli* with 503.09 IU/mL productivity [5]. In Ao et al. [4], neutral protease from *Aspergillus oryzae* Y1 had an optimal pH and temperature of 7.0 and 55 °C, respectively, with the specific activity of 2264.3 U/mg. Moreover, since the culture liquid activity of AsPs protease toward casein was shown to be 150,800 U/mL, the authors imply that expressing fungi protease in fungi producer strain is more effective.

It has been shown that the use of multicopy plasmids with recombinant DNA organized in tandem clusters for transformation of *K. phaffii* is an effective way to increase the production of a target protein [20]. The plasmid pPZL-2xProt_AsPs, developed throughout the research, carries a tandem construct of the optimized gene pAsPs, suitable for expression in *K. phaffii*. The authors assume that using a tandem copy organisation is an effective way of producing high yields of pAsPs protease in bioreactors.

In Ke et al. [9], neutral protease NpI (from *A. oryzae* 3042) heterologously synthesized in *K. phaffii* had a product yield of 43,101 IU/mL and an optimum at pH 8.0 and 55 °C, while the pH range of activity was 5.0 to 9.0; their enzyme retained its activity at 50 °C for 120 min. Furthermore, inhibition by divalent copper ions was demonstrated by Ke et al. with both the recombinant NpI protease [9] and pAsPs. With the activity of AsPs protease being 150,800 U/mL, pH optimum—6.5–8.0, and the temperature optimum—50–60 °C, with the highest activity at 60 °C, the data obtained is comparable to previously published results.

According to the work of Lei et al. [21], who characterized a recombinant NpI obtained from *A. oryzae*, glutamate at position 436 is a part of the active site of the enzyme. By the EMBOSS water algorithm (https://www.ebi.ac.uk/Tools/psa/emboss_water, accessed on 6 November 2022), it was demonstrated here that the sequence identity is 94.9%, while similarity is 96.7%, and the glutamate at position 209—reported to be involved in the active site of *A. oryzae* NpI—is present at the same position in pAsPs. Because active sites of enzymes are extremely conserved regions, our results suggest that the pAsPs protease also belongs to the class of neutral extracellular proteases and has the corresponding properties for industrial application.

During the bioreactor production, microbial growth was seen throughout the whole fermentation process, with a maximum biomass content of 240 g/L. It is important to note that for secreted products of biosynthesis, the concentration of producer cells in the medium is virtually proportional to the amount of secreted protein [22]. For instance, during the production of a neutral NpI protease in *K. phaffii*, Ke et al. [9] reported the highest wet-mass content of 266.4 g/L, which is comparable to the result obtained in our laboratory.

The creation of the strain producing the neutral pAsPs protease for cultivation in a bioreactor is a major step toward subsequent use of this enzyme in industry. For example, according to Deng et al., treatment of the soy isolate that is a protein source for the fish *Carassius* with a recombinant NpI protease provides abundant small peptides and reduces the number of oxidants, thereby significantly improving fish health [23]. It is also important to note that proteases are one of the main components of fabric detergents, hydrolyzing the large protein molecules, thus removing them from the surface of cloth [24]. Considering that pAsPs protease is active towards casein, azocasein, and haemoglobin, both in high pH and temperatures, the authors propose that it is a good candidate for industry application, especially as a detergent component.

To sum up, in our laboratory, a neutral protease of *A. pseudotamarii* was selected (pAsPs), optimized, and then expressed in a strain of the yeast *K. phaffii*. The protein was produced in a 5 L bioreactor with an enzyme yield of 150,800 U/mL of culture liquid in casein terms. It was demonstrated that the resultant enzyme has an activity of 2320 U/mg toward hemoglobin and 25,344 U/mg toward azocasein, making it promising for manufacturing applications, including the food industry.

## 4. Materials and Methods

### 4.1. Strains, Plasmids, and Media

*K. phaffii* strain T07 was chosen as the microbe for the expression of the enzyme. The T07 strain was previously discovered in Simferopol (Russia) and characterized in our laboratory. The T07 strain is deposed at the national bioresource center in an all-Russian collection of industrial microorganisms (VKPM) based at the Kurchatov Institute Research Center (Acsession Number #Y-4936). Plasmids were constructed by means of the *E. coli* XL1-blue strain (Merck Group, Darmstadt, Germany). The pPZL plasmid, based in pPICZa vector (ThermoFisher, Waltham, MA, USA) with modifications, was utilized to create an integration vector and the expression construct. Culture media components were purchased from Difco (Franklin Lakes, NJ, USA), whereas restriction endonucleases, Taq polymerase, T4 DNA ligase, and alkaline phosphatase were purchased from SibEnzyme Ltd. (Novosibirsk, Russia). Q5^®^ High-Fidelity DNA polymerase and the NEBuilder HiFi reagent kit were acquired from New England Biolabs (Ipswich, MA, USA). The primers were synthesized by BioSet Ltd. (Novosibirsk, Russia).

### 4.2. Construction of the Gene for Genomic Integration

During an analysis of databases of genetic sequences from microorganisms, a sequence of a previously unstudied protease of *A. pseudotamarii* (BDV38DRAFT_289492; Gene ID: 43645458) was selected. Next, via the OPTIMIZER algorithm, codon composition of the *A. pseudotamarii* protease gene was optimized [25]. To obtain sequences suitable for expression in *K. phaffii*, signal peptides found in the amino acid sequences by the SignalP-5.0 algorithm [26] were removed. The optimized pAsPs gene for genomic integration in the producer strain was synthesized by Atg:biosynthetics (Germany). The optimized gene accession number is OP183485.

The Gibson assembly method was used to create initial constructs [27]. The primers employed to clone the NpI metalloproteinase genes under the control of the AOX1 gene promoter and terminator were 5′-AGCTTCAGCCTCTCTTTTCTCG-3′, 5′-AGTCGACCATCATCATCATCATC-3′, 5′-AGAAAAGAGAGGCTGAAGCTCATCCAACTCATCACGCCCA-3′, and 5′-GATGATGATGATGGTCGACTTAACATGCGTCGGAAGGAAC-3′.

Amplification of the protease gene was carried out in plasmid pPIC9, whereas amplification of the plasmid part of the construct was conducted in the pPZL plasmid. The amplification cycling conditions were 1 cycle with 95 °C 3 min; 5 cycles with 95 °C 10 s 58 °C 15 s, 72 °C 120 s; 25 cycles with 95 °C 10 s, 63 °C 15 s, 72 °C 120 s; with further storage at 12 °C.

The amplicons were incubated with restriction enzymes *Mal*I, *Bgl*II, and *Bam*HI at 37 °C for 30 min. Plasmid pPZL-Prot_AsPs, containing one copy of AsPs gene, was incubated with *Bgl*II at 37 °C for 30 min. Next, reaction with alkaline phosphatase was performed at 16 °C for 120 min. After that, the fragments were purified on KAPA Pure Beads magnetic particles (Roche, Basel, Switzerland) according to the manufacturer’s instructions. Derived fragments then were ligated using highly active T4 DNA ligase (SibEnzyme, Novosibirsk, Russian Federation) in the volume of 30 μL at 16 °C for 30 min.

Next, the plasmid was isolated from the reaction mixture by means of magnetic beads and used to transform *E. coli* XL1-blue cells. The transformation was performed using electroporation [28]. After the electroporation, the resulting transformants were seeded on an LB agar medium (1% of tryptone, 0.5% of yeast extract, and 2% of agar, pH 8.0) with the addition of zeocin to a final concentration of 20 μg/mL. The resultant cells were incubated in a thermostat at 37 °C for 1 day.

Screening of the transformants for the presence of the DNA correctly inserted into the plasmid was performed by PCR. For this purpose, a pair of primers was utilized: Prot_Apse_to_alpha_F and Prot_Apse_to_6His_R. A colony was transferred to a culture plate with the LB medium and zeocin at 20 μg/mL, and then the loop was dipped in 150 μL of 10 mM Tris-EDTA buffer pH 8 (TE buffer) to prepare a cell suspension. From the 150 μL of the suspension, 50 μL was transferred to 500 μL of the LB medium with zeocin (20 μg/mL), and the sample was incubated at 37 °C. The remaining 100 μL of each suspension was incubated at 98 °C for 2 min, centrifuged at 13,000 rpm, and then used as a PCR template. The PCR program was 1 cycle with 95 °C 3 min; 25 cycles with 95 °C 10 s, 62 °C 15 s, 72 °C 30 s; with further storage at 12 °C.

To select a clone carrying the desired genetic construct, the obtained amplicons were separated by electrophoresis and examined visually.

### 4.3. Preparation of a Plasmid with Two Copies of the Gene

To construct a plasmid with two tandem copies of the target gene, the pPZL-Prot_AsPs plasmid carrying one copy of the optimized pAsPs protease gene of *A. pseudotamarii* was used. Amplification was performed with Q5^®^ High-Fidelity Polymerase and a pair of primers: BglAoxProm_F (5′-atgcatgcAGATCTAACATCCAAAGACGAAAGG-3′) and BamHIAoxTerm_R (5′-atgcatgcGGGATCCGCACAAACGAAGG-3′). The amplification program was 1 cycle with 95 °C 3 min; 5 cycles with 95 °C 10 s, 58 °C 15 s, 72 °C 180 s; 25 cycles with 95 °C 10 s, 63 °C 15 s, 72 °C 80 s; with further storage at 12 °C.

The reaction mixture was incubated with restriction enzyme *Mal*I at 37 °C for 30 min, and then DNA was purified on KAPA Pure Beads magnetic particles (Roche, Basel, Switzerland) according to the manufacturer’s instructions. The resultant DNA and plasmid pPZL-Prot_AsPs were incubated with restriction enzymes *Bgl*II and *Bam*HI at 37 °C for 30 min, after which they were reacted with alkaline phosphatase at 16 °C for 120 min. The fragments purified on the magnetic beads were ligated with the help of a highly active T4 DNA ligase (SibEnzyme) in a 30 μL reaction at 16 °C for 30 min.

For electroporation of competent cells of *E. coli* strain XL1-blue, 1 μL of the reaction mixture was used. The transformants were resuspended in 1 mL of the LB medium, followed by incubation for 45 min at 37 °C. Next, 100 μL of the cell suspension was placed on the LB agar medium supplemented with 20 μg/mL zeocin and incubated at 37 °C for 12 h. Transformants were identified visually by means of the characteristic blue color of the colonies.

### 4.4. Integration of Plasmid pPZL-2xProt_AsPs (Expressing the Protease) into the Genome of K. Phaffii Strain T07

Plasmid pPZL-2xProt_AsPs, which contains two tandem copies of the target gene, was transfected into the *K. phaffii* T07 strain by electroporation. After the electroporation, the transformants were cultured for 2 h at 30 °C in 1 mL of 1 M sorbitol. After that, ⅕ of the resulting cell suspension was seeded on a 1.5% yeast extract–peptone–dextrose (YPD) agar medium (BD Difco) supplemented with glucose and zeocin up to 200 μg/mL.

### 4.5. Evaluation of Enzymatic Activity of the Obtained Strains

The colonies were seeded in 2 mL of yeast extract–peptone–glycerol–methanol (YPgM) supplemented with 0.3% of glucose and 1% of methanol in a 24-deep-well plate, with a single colony per well. The *K. phaffii* T07 strain without the transfected pAsPs protease gene served as a negative control. The cells were incubated at 30 °C in a thermoshaker (480 rpm). Every 24 h, 200 μL of 10% methanol was added into each well of the plate. After 72 h, 500 μL of the cultures were taken from each well of the plate, transferred to 1.5 mL tubes, and the cells were pelleted by centrifugation at 4000× *g* for 5 min.

Enzymatic activity was assayed by means of a change in the color of a solution of a polypeptide bonded with a dye [29]. To this end, 20 μL of each culture liquid was placed into a well of a plate, then 80 μL of a buffer (50 mM Tris-HCl pH 9.0, 1 mM CaCl_2_) and 100 μL of a substrate solution were added. The substrate was prepared by dissolving 50 mg of N-succinyl-Ala-Ala-Pro-Phe-P-nitroaniline in 1 mL of dimethyl sulfoxide to obtain a stock solution; the latter was diluted 45-fold with 0.01% Triton X-100. During the activity assay, a sample of a commercial protease served as a positive control, whereas the supernatant from a culture of *K. phaffii* T07 devoid of the inserted target gene served as a negative control.

### 4.6. Lab Scale Production of the Enzyme in a Bioreactor

The production of the recombinant protease of *A. pseudotamarii* was conducted in a 7.5 L ProLab fermenter (GPC, Paris, France). The chosen clone of *K. phaffii* was plated on the YPD agar medium and cultivated for 48 h to obtain individual colonies. Stand-alone colonies were inoculated individually into 5 mL of the YPD medium containing 200 μg/mL zeocin and cultivated overnight at 30 °C. The overnight culture (4 mL) was inoculated into 400 mL of a culture medium (in four 500 mL flasks, each containing 100 mL of the YNB medium) and cultivated for 48 h in a shaker at 30 °C and 250 rpm.

The initial culture was aseptically introduced (the loop was flame-sterilized) into the 7.5 L bioreactor containing 4 L of a salt medium [32.5 g/L glycerol, 9.375 g/L (NH_4_)_2_SO_4_, 1.875 g/L CaSO_4_∙2H_2_O, 0.9375 g/L NaCl, 3.75 g/L MgSO_4_∙7H_2_O, and 3.75 g/L KH_2_PO_4_]. The bioreactor was set up beforehand and sterilized by autoclaving for 45 min at 121 °C.

At the start of cultivation, the following settings were applied: temperature 30 °C with a constant air flow of 3 L/min and an initial stirrer speed of 400 rpm. Dissolved oxygen was maintained at >20% via a gradual increase in the stirring speed to 1200 rpm. The appearance of the foam was reduced using defoamer (Sigma-Aldrich, St. Louis, MI, USA). The pH of the medium was maintained at 5.8–6.0 by means of a 4 M NaOH solution. Before inoculation, trace elements (2.5 mL/L) and vitamins (2.5 mL/L) were added to the medium (Table 3).

### 4.7. Purification of the Enzyme from the Yeast Culture Liquid

All procedures were carried out at 5 °C ± 2 °C. The culture liquid was separated from cells and other particulate matter by centrifugation at 4000 rpm for 10 min. The supernatant was purified to remove low-molecular-weight impurities and was concentrated on a SartoJet tangential filtration system (Sartorius, Germany) with the help of filters having pore size corresponding to a 10 kDa molecular weight cutoff.

The resulting concentrate was frozen at −70 °C and lyophilized (Labconco, Kansas City, MO, USA). The lyophilized intermediate preparation was dissolved in 10 mM sodium phosphate buffer (pH 7.3), and chromogenic impurities were removed by gel filtration on Sephadex G-25 (Sigma-Aldrich, St. Louis, MI, USA), after which the product was eluted with 10 mM sodium phosphate buffer (pH 7.3). Further purification of the enzyme was performed by ion exchange chromatography on a 10 mL column filled with DEAE-Sepharose 6HF anion exchange resin (Biotoolomics, UK). The column was washed with 10 mM sodium phosphate buffer (pH 7.3), then the protein was eluted via a linear gradient of NaCl (0–0.5 M) in the initial buffer.

Afterward, 2.5 mL fractions were collected. The fractions where the highest proteolytic activity was detected (number 18 to number 22) were pooled, desalted, and concentrated using centrifugal concentrators (10 kDa).

### 4.8. Determination of the Temperature and pH Optima of the Enzyme

Proteolytic activity was quantified by the Kunitz method [30] with cow milk casein (Sigma-Aldrich, St. Louis, MI, USA). To determine optimal pH, a 50 mM Tris-HCl buffer was employed for the pH range 6.0–7.0, whereas for the 7.5–11.0 pH range, 50 mM Tris-glycine buffer was utilized. The protein concentration was measured by the Bradford assay [31]. Test and control samples were incubated at 55 °C for 20 min, and then the reaction was stopped by the addition of 1 mL of 1.2 M trichloroacetic acid (TCA). Then, the solution was centrifuged at 12,000 rpm for 10 min at 2 °C. The optical density of the hydrolysis products in the supernatants was determined at λ = 275 nm.

After the pH optimum was found, the temperature optimum of the protease was identified. A 2% casein solution in the optimal buffer was heated at various temperatures, and a required amount of the enzyme solution at 0.06 mg/mL in the same buffer was added to the heated substrate solution. Test and control samples were incubated at a given temperature for 20 min, and then the reaction was stopped by adding 1 mL of 1.2 M TCA. Next, the solution was centrifuged at 12,000 rpm for 10 min at 2 °C. The optical density of the hydrolysis products in the supernatants was quantitated at λ = 275 nm.

The enzyme solution was purified via removal of impurities by means of deionized water cooled to 2 °C in Vivaspin centrifuge concentrators (Sartorius Stedim Biotech, Göttingen, Germany) with membrane pore size corresponding to the 10 kDa molecular weight cutoff. The enzyme concentrate in the water was diluted with a 50 mM Tris-glycine buffer of certain pH so that the protein concentration in the solution was 0.06 mg/mL. Test and control samples were incubated at a given temperature for 20 min, and then the reaction was stopped by the addition of 1 mL of 1.2 M TCA. Finally, the solution was centrifuged at 12,000 rpm for 10 min at 2 °C. The optical density of the hydrolysis products in the supernatants was determined at λ = 275 nm.

### 4.9. Characterization of Enzyme Inhibition by Metal Ions at High Concentrations

To determine the effect of metal ions on the proteolytic activity of the enzyme, 0.4 mL of a 2% casein solution in 25 mM Tris-HCl buffer (pH 7) containing either 3 or 15 mM of a given salt was heated to 60 °C, and 0.2 mL of the enzyme solution was introduced in the same buffer with a salt concentration of either 3 or 15 mM; the protein concentration in the reaction mixture was 0.02 mg/mL [32,33]. The reaction mixture was incubated at 60 °C for 20 min, and the enzymatic reaction was stopped by adding 1 mL of 1.2 M TCA. Control substrate samples (without the enzyme) containing the corresponding concentration of a given salt were heated at the same temperature. After 20 min, 1 mL of 1.2 M TCA and 0.2 mL of the enzyme solution containing metal ions were introduced into the control samples. The resulting precipitates were separated by centrifugation at 12,000 rpm for 10 min at 2 °C. The optical density of the hydrolysis products in the supernatants was measured at λ = 275 nm.

### 4.10. Determination of Proteolytic Activity toward Azocasein

To quantify the proteolytic activity, 0.8 mL of a 0.2% azocasein solution in a 25 mM Tris-glycine buffer (pH 7.2) and 0.4 mL of the enzyme solution with a protein concentration of 0.04 mg/mL in the same buffer were mixed and incubated in a water bath at 60 °C for 5 min. The reaction was stopped by adding an equal volume of 1.2 M TCA. To set up a negative control reaction, 0.8 mL of a 0.2% azocasein solution in 25 mM Tris-glycine buffer (pH 7.2) was incubated under the same conditions without the enzyme. Afterward, 0.4 mL of 1.2 M TCA and 0.4 mL of a 0.04 mg/mL enzyme solution in the same buffer were introduced. The samples were centrifuged for 10 min at 12,000 rpm and 5 °C, and absorbance in the supernatant was determined at λ = 340 nm. One unit of activity was assumed to be the amount of protease causing a change in absorbance by one unit of absorbance in 1 h [32,33,34].

### 4.11. An Assay of Proteolytic Activity toward Hemoglobin

To determine the proteolytic activity toward hemoglobin as a substrate, we used the assay of pepsin A activity [35] with our modifications. For this purpose, 0.3 mL of 2% bovine serum hemoglobin (Sigma-Aldrich, Germany) in 10 mM sodium phosphate buffer (pH 7.2) supplemented with 0.9% NaCl was incubated at 60 °C for 5 min. Then, 0.1 mL of the enzyme solution in the same buffer at 0.04 mg/mL was added to the heated substrate. After incubation in a water bath at 60 °C for 10 min, the reaction was stopped with 0.6 mL of 0.3 M TCA. As a negative control, 0.3 mL of a 2% hemoglobin solution in the same buffer (pH 7.2) was employed, which was incubated in a water bath at 60 °C for 5 min without the enzyme solution. Next, 0.6 mL of 0.3 M TCA and 0.1 mL of the 0.04 mg/mL enzyme solution in the same buffer were added. The samples were centrifuged for 10 min at 12,000 rpm and 5 °C, and absorbance in the supernatant was measured at λ = 280 nm.

### 4.12. Enzyme Stability under the Optimal Conditions

To prepare a 2% casein solution, 4 g of casein was dissolved via alkalization with 2 M NaOH in 100 mL of purified water. The solution of casein in water was diluted twofold with 50 mM Tris-glycine buffer (pH 7.0). Subsequently, this solution served as a substrate. The enzyme solution was diluted with 50 mM Tris-glycine buffer (pH 7.0) to a concentration of 0.08 mg/mL. Proteolytic activity was determined by the Kunitz method [32] as described above.

### 4.13. Electrophoretic Analysis

Electrophoretic analysis was performed according to Laemmli assay [34]. For this analysis, 2 mg of a lyophilized sample was dissolved in 1 mL of a buffer (62.5 mM Tris-HCl pH 6.8, 2% of sodium dodecyl sulfate, and 1% of phenylmethylsulfonyl fluoride) and was sonicated on ice for 3 min (two pulses with 2 s intervals) by means of an ultrasonicator at 91 W power (Cole-Parmer Instrument, Vernon Hills, IL, USA). Next, 10 μL of a sample was mixed with loading buffer (62.5 mM Tris-HCl pH 6.8, 25% of glycerol, 2% of SDS, 0.01% of bromophenol blue, and 5% of mercaptoethanol) at a ratio of 1:2 and incubated at 100 °C for 5 min. Then, 20 μL of each sample was introduced into a gel well and concentrated in a 4% polyacrylamide gel containing SDS (SDS-PAAG; acrylamide/bisacrylamide ratio 37.5:1.0, 0.1% of SDS, 0.125 mM Tris-HCl pH 6.8, 0.1% of tetramethylethylenediamine, and 0.05% of ammonium persulfate) at a current of 15 mA in a Mini-PROTEAN^®^ Tetra Cell electrophoretic unit (Bio-Rad, Hercules, CA, USA). Next, the proteins were separated in an SDS-PAGE (acrylamide/bisacrylamide ratio 37.5:1.0, 0.1% of SDS, 0.375 mM Tris-HCl pH 8.8, 0.05% of TEMED, and 0.05% of ammonium persulfate) at a current of 25 mA in the same electrophoretic unit. The gel was stained with Sypro Ruby and visualized using a VersaDoc MP4000 gel documentation system (Bio-Rad, Hercules, CA, USA).

### 4.14. Mass Spectrometry

For protein identification, a sample of a lyophilized culture fluid was dissolved in 100 mM NH_4_HCO_3_ to 5 mg/mL. Next, a 10 μL sample was incubated with 190 μL of reduction/reconstitution buffer [20 mM tris(2-carboxyethyl)phosphine, 40 mM 2-(2-(4,8-dimethyl-7-((3-methylbut-2-en-1-yl)oxy)-2-oxo-2H-chromen-3-yl) acetamido)acetic acid, 100 mM NH_4_HCO_3_, and 0.1% of sodium deoxycholate (SDC)] for 5 min at 4 °C, then for 5 min at 90 °C, and after that, for 20 min in the dark at room temperature. Trypsin was added at a ratio of 1:100, and the mixture was kept overnight at 37 °C. The reaction was stopped with 1% trifluoroacetic acid (TFA). SDC was removed from the samples through emulsification with an equal volume of ethyl acetate 3 times for 5 min, with the removal of the organic phase. For the third extraction, ethyl acetate with 1% of TFA was applied [35]. Next, a sample of the solution (volume corresponding to 20 μg of protein) was purified by means of StageTips according to Rappsilber et al. [36], i.e., the sample was concentrated by solid-phase extraction in a C18 cartridge and then reconstituted at the first step of the gradient for ultra-high-performance liquid chromatography. After elution, the samples were dried in a rotary evaporator and stored at −20 °C. For protein identification, each sample was dissolved in 20% acetonitrile with 0.1% of TFA to a protein concentration of 1 μg/μL and applied to a mass spectrometer target in a mixture with 20 mg/mL 2,5-dihydroxybenzoic acid, 0.1% of TFA, and 70% of acetonitrile.

Tandem mass spectrometry was conducted on an UltraFlex III MALDI tandem time-of-flight mass spectrometer (Bruker, Billerica, MA, USA). The Mascot software database, compiled from theoretical enzyme sequences, was utilized to identify the pAsPs protease in accordance with its optimized theoretical sequence.

## 5. Conclusions

For the first time in our laboratory, the neutral protease pAsPs of *A. pseudotamarii* was optimised and cloned into *K. phaffii* yeast strain, a neutral protease pAsPs of *A. pseudotamarii*. The product was produced in a 5 L bioreactor with an enzyme yield of 150,800 U/mL of cultural liquid on casein. It was shown that the obtained enzyme had an activity of 2320 U/mg for hemoglobin and 25,344 U/mg for azocasein, which makes it promising for use in industry, including food production.

## Figures and Tables

**Table 1:** The matched peptide masses of the pAsPs protease.

Peptide	Observed Molecular Mass	Expected Molecular Mass	Calculated Molecular Mass	Individual Ions Scores
K.LVVDGMALQPCNPNCIQAR.D + 2 Carbamidomethyl (C); Deamidated (NQ)	2156.3726	2155.3653	2156.0177	59
R.CLNALESGGMGEGWGDFMATAIR.L + Carbamidomethyl (C)	2443.4949	2442.4876	2442.0766	49
K.GGAGNDYVILNAQDGSGTNNANFATPPDGQPGR.M + Deamidated (NQ)	3245.9478	3244.9405	3245.4610	(128)
R.GLGEGAEYHASR.R	1246.7783	1245.7710	1245.5738	32
R.LKSGDTHSTDYTMGEWAANR.K + Deamidated (NQ)	2240.4229	2239.4156	2239.9804	51
R.CLNALESGGMGEGWGDFMATAIR.L + Carbamidomethyl (C); Deamidated (NQ); Oxidation (M)	2459.5244	2458.5171	2459.0556	(41)

**Table 2:** Levels of enzymatic activity in the presence of various salts.

Salt	Proportion of Initial Activity (%)	
	3 mM Salt Concentration	15 mM Salt Concentration
25 mM Tris-HCl pH 7.0	100	100
KCl	79 ± 2	95 ± 2
NaCl	93 ± 2	93 ± 2
CaCl_2_	67 ± 2	38 ± 2
ZnCl_2_	19 ± 2	9 ± 2
BaCl_2_⋅2H_2_O	58 ± 2	30 ± 2
MgCl_2_⋅6H_2_O	81 ± 2	54 ± 2
MnCl_2_⋅5H_2_O	21 ± 2	9 ± 2
Pb(NO_3_)_2_	14 ± 2	5 ± 2
CuCl_2_	0	0
CoCl_2_⋅6H_2_O	21 ± 2	20 ± 2

**Table 3:** Concentrations of trace elements and vitamins.

Substance	Amount per 1000 mL
Copper sulfate pentahydrate (CuSO_4_∙5H_2_O)	3 g
Sodium iodide (NaI)	0.4 g
Manganese sulfate (MnSO_4_)	2 g
Sodium molybdate dihydrate (Na_2_MoO_4_∙2H_2_O)	1 g
Boric acid (H_3_BO_3_)	0.1 g
Cobalt chloride hexahydrate (CoCl_2_∙6H_2_O)	0.5 g
Ferrous sulfate heptahydrate (FeSO_4_∙7H_2_O)	33 g
Sulfuric acid (H_2_SO_4_)	5 mL
Zinc sulfate (ZnSO_4_∙7H_2_O)	5 g
Biotin	0.0508 g
Calcium pantothenate	0.2 g
Folic acid	0.01 g
Inositol	1 g
Niacin	0.2 g
p-Aminobenzoic acid	0.1 g
Pyridoxine hydrochloride	0.2 g
Riboflavin	0.1 g
Thiamine hydrochloride	0.2 g

## Data Availability

Not applicable.

## References

[1] Martínez-Medina G.A., Barragán A.P., Ruiz H.A., Ilyina A., Hernández J.L.M., Rodríguez-Jasso R.M., Hoyos-Concha J.L., Aguilar-González C.N. (2019). Fungal Proteases and Production of Bioactive Peptides for the Food Industry. Enzymes in Food Biotechnology.

[2] Naeem M., Manzoor S., Abid M.-U., Tareen M.B.K., Asad M., Mushtaq S., Ehsan N., Amna D., Xu B., Hazafa A. (2022). Fungal Proteases as Emerging Biocatalysts to Meet the Current Challenges and Recent Developments in Biomedical Therapies: An Updated Review. J. Fungi.

[3] Madhavan A., Arun K., Binod P., Sirohi R., Tarafdar A., Reshmy R., Awasthi M.K., Sindhu R. (2021). Design of novel enzyme biocatalysts for industrial bioprocess: Harnessing the power of protein engineering, high throughput screening and synthetic biology. Bioresour. Technol..

[4] Ao X.-L., Yu X., Wu D.-T., Li C., Zhang T., Liu S.-L., Chen S.-J., He L., Zhou K., Zou L.-K. (2018). Purification and characterization of neutral protease from *Aspergillus oryzae* Y1 isolated from naturally fermented broad beans. AMB Express.

[5] Hu Y., Li T., Tu Z., He Q., Li Y., Fu J. (2020). Engineering a recombination neutral protease I from *Aspergillus oryzae* to improve enzyme activity at acidic pH. RSC Adv..

[6] Shah M.A., Mir S.A., Paray M.A. (2014). Plant proteases as milk-clotting enzymes in cheesemaking: A review. Dairy Sci. Technol..

[7] Shafee T. (2014). Evolvability of a Viral Protease: Experimental Evolution of Catalysis, Robustness and Specificity. Doctoral Dissertation.

[8] Shu M., Shen W., Yang S., Wang X., Wang F., Wang Y., Ma L. (2016). High-level expression and characterization of a novel serine protease in *Pichia pastoris* by multi-copy integration. Enzym. Microb. Technol..

[9] Ke Y., Huang W.-Q., Li J.-Z., Xie M.-Q., Luo X.-C. (2012). Enzymatic Characteristics of a Recombinant Neutral Protease I (rNpI) from *Aspergillus oryzae* Expressed in Pichia pastoris. J. Agric. Food Chem..

[10] Guo J.-P., Ma Y. (2008). High-level expression, purification and characterization of recombinant *Aspergillus oryzae* alkaline protease in Pichia pastoris. Protein Expr. Purif..

[11] Guangbo Y., Min S., Wei S., Lixin M., Chao Z., Yaping W., Zunxi H. (2021). Heterologous expression of nattokinase from *B. subtilis* natto using *Pichia pastoris* GS115 and assessment of its thrombolytic activity. BMC Biotechnol..

[12] Sandhya C., Sumantha A., Szakacs G., Pandey A. (2005). Comparative evaluation of neutral protease production by *Aspergillus oryzae* in submerged and solid-state fermentation. Process Biochem..

[13] Ito Y., Peterson S.W., Wicklow D.T., Goto T. (2001). *Aspergillus pseudotamarii*, a new aflatoxin producing species in Aspergillus section Flavi. Mycol. Res..

[14] Heistinger L., Gasser B., Mattanovich D. (2020). Microbe Profile: *Komagataella phaffii*: A methanol devouring biotech yeast formerly known as *Pichia pastoris*. Microbiology.

[15] Kozlowski L.P. (2016). IPC—Isoelectric Point Calculator. Biol. Direct..

[16] Rozanov A.S., Voskoboev M.E., Bogacheva N.V., Korzhuk A.V., Shlyahtun V.N., Mescheryakova I.A., Romancev V.A., Bochkov D.V., Zadorozhnyy A.V., Peltek S.E. (2021). Cloning and expression of a cellulase gene from *Penicillium* sp. ‘occitanis’ in *Komagataella phaffii* T07. Vavilovskii Zhurnal Genet. Sel..

[17] Braun-Galleani S., Dias J.A., Coughlan A.Y., Ryan A.P., Byrne K.P., Wolfe K.H. (2019). Genomic diversity and meiotic recombination among isolates of the biotech yeast *Komagataella phaffii* (*Pichia pastoris*). Microb. Cell Factories.

[18] Bernauer L., Radkohl A., Lehmayer L.G.K., Emmerstorfer-Augustin A. (2021). *Komagataella phaffii* as emerging model organism in fundamental research. Front. Microbiol..

[19] Ata Ö., Ergün B.G., Fickers P., Heistinger L., Mattanovich D., Rebnegger C., Gasser B. (2021). What makes *Komagataella phaffii* non-conventional?. FEMS Yeast Res..

[20] Lopes T.S., Hakkaart G.-J.A., Koerts B.L., Raué H.A., Planta R.J. (1991). Mechanism of high-copy-number integration of pMIRY-type vectors into the ribosomal DNA of Saccharomyces cerevisiae. Gene.

[21] Lei D., Xu Y., He Q., Pan Y., Chen B., Xiong L., Li Y. (2013). Glycosylation analysis of recombinant neutral protease I from *Aspergillus oryzae* expressed in Pichia pastoris. Biotechnol. Lett..

[22] Duman-Özdamar Z.E., Binay B. (2021). Production of industrial enzymes via *Pichia pastoris* as a cell factory in bioreactor: Current status and future aspects. Protein J..

[23] Deng J.-J., Shi D., Zhao M., Li Z., Lu D., Xu S., You Z., Li J., Luo X. (2021). Recombinant neutral protease rNpI as fish feed additive to improve protein digestion and growth. Aquac. Res..

[24] Naveed M., Nadeem F., Mehmood T., Bilal M., Anwar Z., Amjad F. (2021). Protease—A Versatile and Ecofriendly Biocatalyst with Multi-Industrial Applications: An Updated Review. Catal. Lett..

[25] Puigbò P., Guzmán E., Romeu A., Garcia-Vallvé S. (2007). OPTIMIZER: A web server for optimizing the codon usage of DNA sequences. Nucleic Acids Res..

[26] Nielsen H., Tsirigos K.D., Brunak S., von Heijne G. (2019). A Brief History of Protein Sorting Prediction. J. Protein Chem..

[27] Gibson D.G., Young L., Chuang R.-Y., Venter J.C., Hutchison C.A., Smith H.O. (2009). Enzymatic assembly of DNA molecules up to several hundred kilobases. Nat. Methods.

[28] Young J.L., Dean D.A. (2015). Electroporation-mediated gene delivery. Adv. Genet.

[29] Bradford M.M. (1976). A rapid and sensitive method for the quantitation of microgram quantities of protein utilizing the principle of protein-dye binding. Anal. Biochem..

[30] Kunitz M. (1947). Crystalline soybean trypsin inhibitor: II. General properties. J. Gen. Physiol..

[31] Bisswanger H. (2019). Practical Enzymology.

[32] Tsvetkov V.C. (1987). Purification and properties of intracellular proteinase E of the flavinogenic fungus *Eremothecium ashbyii*. Biotechnol. Agric. For..

[33] Debananda S., Kshetri N., Kshetri P. (2010). A Thermostable Alkaline Protease from a Moderately Halo-alkalithermotolerant *Bacillus subtilis* Strain SH1. Aust. J. Basic Appl. Sci..

[34] Laemmli U.K. (1970). Cleavage of Structural Proteins during the Assembly of the Head of Bacteriophage T4. Nature.

[35] Gaertner H.F., Puigserver A.J. (1992). Increased activity and stability of poly(ethylene glycol)-modified trypsin. Enzym. Microb. Technol..

[36] Rappsilber J., Mann M., Ishihama Y. (2007). Protocol for micro-purification, enrichment, pre-fractionation and storage of peptides for proteomics using StageTips. Nat. Protoc..

